# A Case of Infectious Mononucleosis With Peritonsillar Abscess in a Middle-Aged Patient

**DOI:** 10.7759/cureus.95772

**Published:** 2025-10-30

**Authors:** Shuya Tatsuki, Ko Kato, Yukinori Tsuruta, Masaki Hayama

**Affiliations:** 1 Otolaryngology, Hyogo Prefectural Nishinomiya Hospital, Nishinomiya, JPN

**Keywords:** abscess drainage, acute tonsillitis, epstein-barr virus, infectious mononucleosis, laryngeal edema, middle-aged, oropharyngeal infection, peritonsillar abscess, secondary bacterial infection

## Abstract

Infectious mononucleosis (IM) is common among young adults but often presents with nonspecific symptoms in middle-aged and older individuals. Cases of IM complicated by peritonsillar abscess (PTA) are uncommon. We report the case of a 41-year-old woman who presented to a local clinic with a sore throat and was initially diagnosed with acute tonsillitis. She was referred to our department after unsuccessful antibiotic and steroid therapy. Because of progressive difficulty with oral intake and laryngeal edema observed on flexible laryngoscopy, she was admitted for close monitoring and supportive care. Epstein-Barr virus (EBV) serology confirmed primary EBV-related IM. On the third hospital day, contrast-enhanced computed tomography (CT) was performed owing to a marked increase in C-reactive protein (CRP) levels, revealing a right-sided PTA. Aspiration drainage yielded oral commensal flora, and the patient responded favorably to antibiotic therapy. The patient improved clinically following drainage and antibiotic therapy and was discharged without recurrence. Even after a diagnosis of IM is established, secondary bacterial infection may occur, leading to PTA; therefore, vigilant clinical monitoring throughout the disease course is essential.

## Introduction

Infectious mononucleosis (IM), most often caused by primary Epstein-Barr virus (EBV) infection, typically affects adolescents and young adults. In middle-aged individuals, the presentation is often atypical, with liver dysfunction and prolonged fever more prominent than the classic triad of pharyngitis, lymphadenopathy, and splenomegaly, making the diagnosis challenging [1]. IM is a generally self-limiting disease, and so antibiotics are unnecessary.

Peritonsillar abscess (PTA) most commonly arises as a complication of acute bacterial tonsillitis [2]. Although uncommon, PTA may also occur in the setting of IM, likely related to transient mucosal immune suppression that facilitates invasion by oral commensal flora [3]. PTA is a potentially serious condition that can cause airway obstruction or spread to deep neck spaces if not promptly treated. Therefore, intensive therapy, including antibiotics and aspiration, is necessary. Early EBV serology, combined with contrast-enhanced computed tomography (CT) and culture when PTA is suspected, enables timely diagnosis and targeted therapy. Delayed recognition may lead to disease progression, airway compromise, or deep neck space infection, underscoring the need for prompt evaluation and intervention.

We present a case of primary EBV-associated IM complicated by PTA in a middle-aged patient. This report highlights the importance of early EBV serology and prompt imaging in the setting of clinical deterioration, along with timely antimicrobial therapy to address potential secondary bacterial infection, and appropriate surgical management, such as needle aspiration or incision and drainage, when indicated. The objective of this report is to describe the clinical course, diagnostic confirmation, and targeted management of EBV-associated IM complicated by PTA in a middle-aged patient.

## Case presentation

A 41-year-old woman developed a sore throat and presented to a local otolaryngology clinic the following day. She was diagnosed with acute tonsillitis and prescribed oral amoxicillin (750 mg/day) and prednisolone (20 mg/day). Despite treatment, her throat pain worsened, and on day 5 after symptom onset, she was referred to our department for further evaluation.

**Figure 1:**
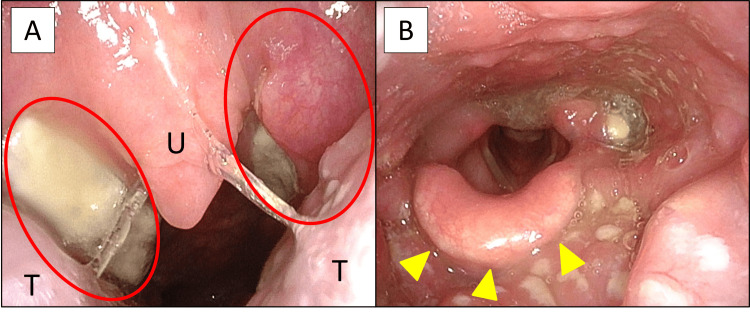
Initial physical and endoscopic findings A: Bilateral tonsillar enlargement with white exudate (circles). B: Endoscopic view showing mild epiglottic edema (arrow heads). U: uvula, T: tongue

She had no significant past medical history, no known allergies, and reported no alcohol consumption or smoking. At presentation, physical examination revealed bilateral tonsillar enlargement with tenderness in the upper neck, and endoscopy demonstrated white tonsillar exudates involving the palatine and lingual tonsils, as well as mild edema of the epiglottis (Figure 1A, B). Blood tests indicated a white blood cell count of 16,600/µL, neutrophils 49.5%, atypical lymphocytes 10.5%, C-reactive protein (CRP) 7.25 mg/dL, aspartate aminotransferase 107 U/L, alanine aminotransferase 149 U/L, γ-glutamyl transpeptidase 98 U/L, and lactate dehydrogenase 519 U/L (Table 1). Because of progressive difficulty with oral intake and laryngeal edema observed on flexible laryngoscopy, she was admitted for close monitoring and supportive care.

**Table 1:** Blood test results during hospitalization WBC: white blood cell, CRP: C-reactive protein, AST: aspartate aminotransferase, ALT: alanine aminotransferase, γ-GT: γ-glutamyl transpeptidase

Test items	Day 1	Day 3	Day 8	Reference range
WBC (/μL)	16,600	12,000	6,800	3300-8600
Neutrophils (%)	49.5	45.5	26.5	28-78
Atypical lymphocytes (%)	10.5	4.0	2.5	0.0-0.0
CRP (mg/dL)	7.25	14.78	2.49	0.00-0.50
AST (U/L)	107	82	129	13-30
ALT (U/L)	149	131	106	7-23
γ-GT (U/L)	98	96	77	9-32
Lactate dehydrogenase (U/L)	519	272	283	124-222

**Figure 2:**
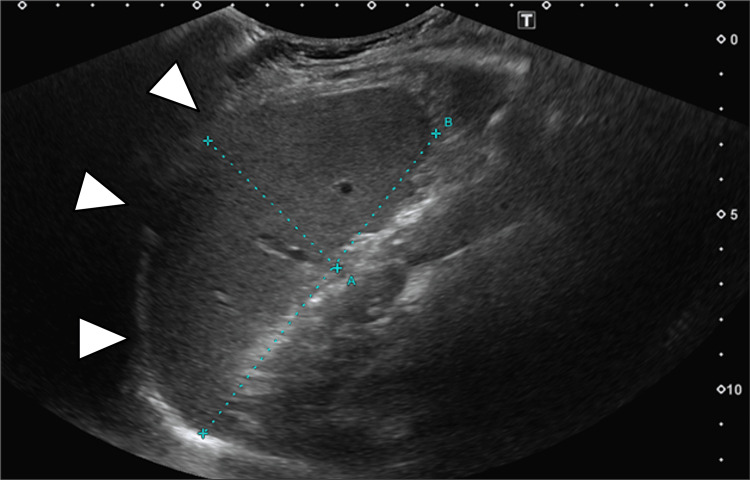
Abdominal Ultrasonography at Initial Presentation Abdominal ultrasonography at the initial presentation revealed mild splenomegaly, with the spleen measuring 109×52 mm (arrow heads). In Japanese adults, the normal splenic length on ultrasonography is generally ≤100 mm.

**Figure 3:**
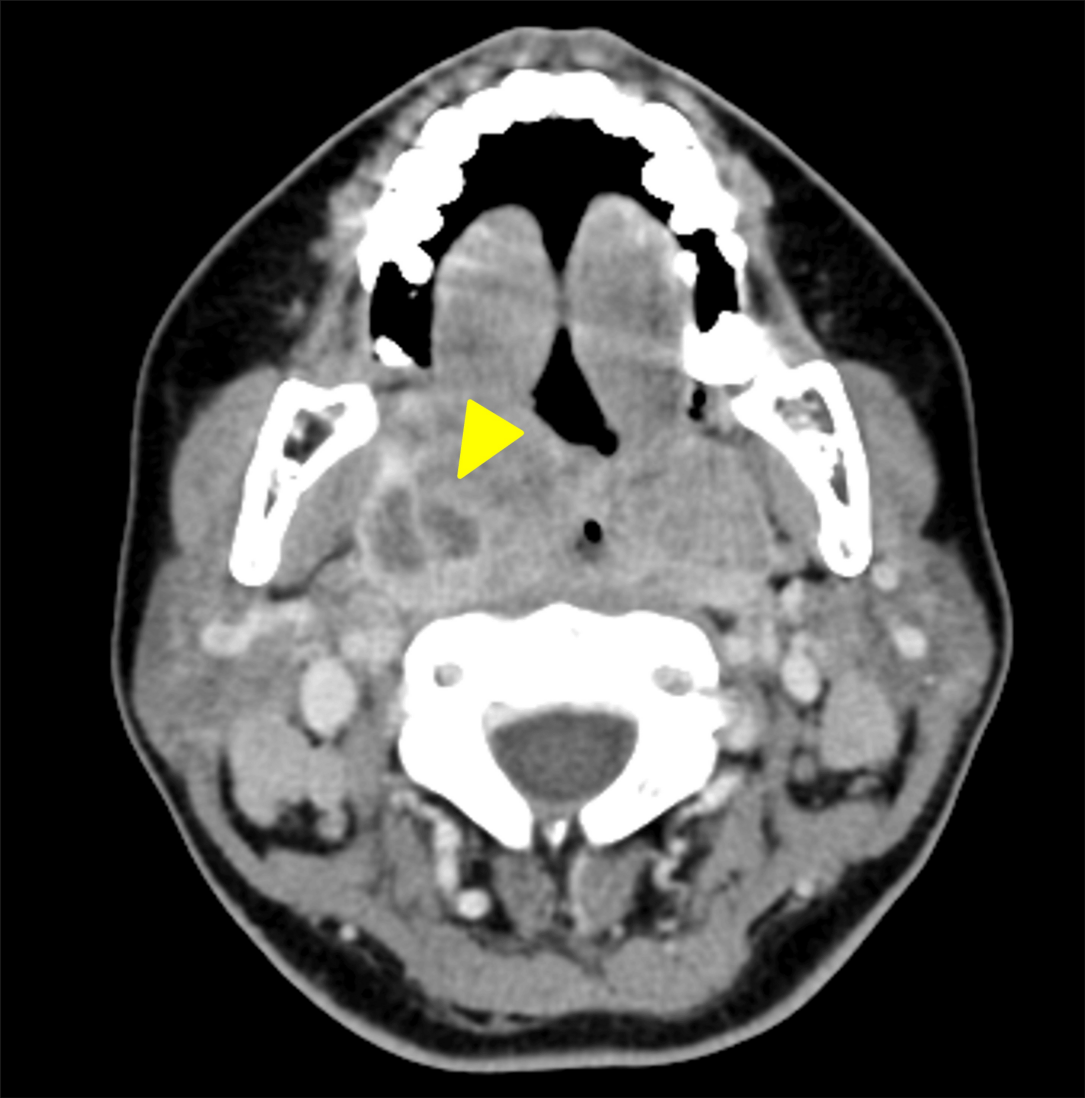
Contrast-enhanced CT on hospital day 3 Axial neck image demonstrating a right peritonsillar abscess (arrow head) with a 13-mm hypodense lesion adjacent to the right tonsil. CT: computed tomography

Based on the admission findings, viral tonsillitis such as IM was suspected, and antibiotics were withheld. Laryngeal edema was treated with intravenous prednisolone (50 mg on hospital day 1 and 20 mg on hospital day 2). Laboratory tests confirmed elevated liver enzymes (Table 1), and abdominal ultrasound revealed mild splenomegaly (Figure 2). On hospital day 3, although the laryngeal edema had improved, the patient developed worsening throat pain with mild trismus and peritonsillar swelling. Blood tests revealed leukocytosis, with a white blood cell (WBC) count of 12,000/μL and neutrophil comprising 45.5%. CRP further increased to 14 mg/dL, raising concern for secondary bacterial infection (Table 1). Contrast-enhanced computed tomography (CT) revealed enlargement of the right tonsil with a 13-mm hypodense lesion along its lateral aspect (Figure 3). Aspiration of the lateral tonsil yielded 0.5 mL of pus. The culture results of the aspirated pus are shown in Table 2. Antibiotic therapy with ceftriaxone (2 g/d) and metronidazole (1500 mg/d) was initiated as the patient had previously tolerated oral amoxicillin without adverse reactions. More than a week had elapsed since the onset of IM, and intensified treatment was required for the concomitant peritonsillar abscess (PTA). The patient was informed of the potential risks before initiating therapy. Viral antibody titers (Table 3) confirmed a diagnosis of IM due to primary EBV infection. Symptoms and laboratory values improved (WBC: 6800 /μL, neutrophils: 26.5%, CRP: 2.49 mg/dL), and antibiotics were discontinued on hospital day 8 (Table 1). She was discharged on day 10 without recurrence. At outpatient follow-up, the patient’s symptoms had fully resolved, with no evidence of recurrent abscess or ongoing hepatic dysfunction.

**Table 2:** Culture results from aspirated pus Culture of pus aspirated from the right peritonsillar abscess, yielding oral commensal flora including the *Streptococcus anginosus* group and anaerobic bacteria.

Organism	Quantity	Characteristics / Clinical significance
*Streptococcus anginosus *group	3+	Oral commensal; strong tendency to form abscesses
Coagulase-negative *Staphylococcus*	Few	Skin commensal; limited significance
Prevotella / Porphyromonas	3+	Anaerobic Gram-negative rods; oral commensals, often associated with abscesses

**Table 3:** Serological viral antibody titers Serological viral antibody titers confirming primary Epstein-Barr virus (EBV) infection, with VCA IgM positivity, elevated VCA IgG, and Epstein-Barr nuclear antigen (EBNA) negativity VCA: viral capsid antigen; VZV: varicella zoster virus; CMV: cytomegalovirus; HSV: herpes simplex virus; Ig: immunoglobulin; CLIA: Clinical Laboratory Improvement Amendments.

Antibody	Initial test	2 weeks later	Reference range	Interpretation
EBV VCA IgM	10	-	<10	Positive
EBV VCA IgG	320	-	<10	Positive
EBV anti-EBNA	≤10	-	<10	Negative
VZV IgM	3.6	2.71	<0.8	Positive
VZV IgG	93.7	102.5	<2.0	Positive
HSV IgM	0.21	-	<0.8	Negative
HSV IgG	14.9	-	<2.0	Positive
CMV IgM (CLIA)	<0.85	-	<0.85	Negative
CMV IgG (CLIA)	≤6.0	-	<6.0	Negative

## Discussion

IM is a viral illness most often caused by primary infection with EBV. It is commonly seen in adolescents and young adults after puberty, with a peak incidence between the ages of 15 and 24 years [4-7]. However, uncommon cases of onset in middle age or later have been reported. In developed countries, where the number of EBV antibody-positive individuals is decreasing, epidemiological data indicate that the age of initial EBV infection is increasing [1,8]. For example, one study reported that only 0.95% (107 cases) of 11,212 individuals diagnosed with IM were aged 40 years or older, although diagnoses have been described even in patients in their 70s and 80s, underscoring the need for continued clinical vigilance [9]. In middle-aged patients, the classic symptoms of pharyngitis, splenomegaly, and lymphadenopathy may be absent or less pronounced [10]. Instead, prolonged fever, fatigue, and liver dysfunction often dominate the clinical picture [10]. The patient in this case was 41 years old, relatively older compared with the typical age of IM onset. At admission, she exhibited elevated aspartate aminotransferase (AST) and alanine aminotransferase (ALT) levels, 10.5% atypical lymphocytes, and mild splenomegaly. She also had a fever (38.2°C) and mild cervical lymphadenopathy. Together with VCA IgM positivity, a high VCA IgG titer, and Epstein-Barr nuclear antigen (EBNA) negativity, these findings established the diagnosis of primary EBV infection. In the early disease stages, classic symptoms such as sore throat, tonsillar enlargement, lymphadenopathy, and hepatosplenomegaly may be absent. Therefore, when middle-aged patients present with unexplained fever or fatigue accompanied by liver dysfunction or atypical lymphocytosis, IM should be considered in the differential diagnosis.

Although PTA has traditionally been described as a rare complication of IM, an increasing number of reports suggest that its frequency may be higher than previously recognized [11-13]. Some studies indicate that PTA occurs in more than 10% of IM cases, and others suggest that bilateral PTA may be more prevalent in patients with severe IM [3,14]. The formation of PTA in IM is thought to involve reduced mucosal antibody production during acute EBV infection, which facilitates adherence and invasion of the tonsillar epithelium by oral commensal flora, ultimately leading to abscess development [15].

From a microbiological perspective, group A *Streptococcus* is not always the primary pathogen, and the isolation of oral commensal flora has significant clinical implications [3]. In this case, laryngeal edema improved following steroid administration; however, CRP increased from 7.25 to 14 mg/dL. Although this rise may have reflected an inflammatory response to EBV itself, the possibility of secondary bacterial infection was considered. Contrast-enhanced CT revealed a 13-mm nonenhancing lesion consistent with an abscess surrounding the right tonsil, which was subsequently drained by needle aspiration. Bacterial cultures yielded oral commensal flora, including the *Streptococcus anginosus* group and *Prevotella*/*Porphyromonas* species. The patient’s condition improved rapidly with treatment consisting of ceftriaxone and metronidazole.

PTA should be suspected in IM patients with poor response to supportive therapy, persistent or worsening symptoms, or rising inflammatory markers. Specifically, clinical features such as trismus, uvular deviation, progressive dysphagia, and elevated CRP should prompt further evaluation with contrast-enhanced CT. If imaging demonstrates a low-attenuation lesion with rim enhancement consistent with abscess formation, aspiration or incision and drainage should be promptly undertaken. Antibiotic regimens should be designed to provide coverage against the *S. anginosus* group and anaerobes. A combination of third-generation cephalosporins with anaerobic coverage is generally appropriate, with subsequent adjustments guided by culture results. When there is a risk of airway compromise, timely airway assessment and expedited drainage are essential [2].

EBV is a herpesvirus. During acute EBV infection, transient IgM positivity or cross-reactivity with other herpesviruses has been reported due to strong immune activation [1,16]. In this case, VZV IgM was positive despite no clinical evidence of varicella-zoster infection, such as rash or neurological symptoms. The mechanism of VZV IgM false positivity is not fully understood but is presumed to result from immune activation from prior infection or assay cross-reactivity [1]. A single IgM measurement is insufficient for diagnosis; consistency with the clinical picture and confirmation with paired serum samples two to three weeks later are required when necessary [1,4,16]. In this case, EBV serology, peripheral blood results, and imaging were consistent, and no changes were observed at the two-week follow-up. Thus, the VZV IgM was considered a nonspecific reaction.

This case adds to the limited reports of EBV-associated IM complicated by PTA in middle-aged adults and emphasizes the diagnostic value of early EBV serology when clinical deterioration occurs. This study has some limitations, including the single-patient design and limited follow-up, which constrain generalizability.

## Conclusions

This case demonstrates that Epstein-Barr virus-associated IM should be considered in middle-aged patients with atypical symptoms such as prolonged fever and hepatic dysfunction. The patient’s symptoms resolved following drainage and targeted antibiotics, and she was discharged on hospital day 10 without recurrence. In cases of clinical deterioration, early EBV serology, contrast-enhanced CT, and timely drainage of PTA are crucial for accurate diagnosis, prevention of complications, and appropriate treatment.
